# Does etiology really matter for epilepsy surgery outcome?

**DOI:** 10.1111/bpa.12965

**Published:** 2021-07-01

**Authors:** Lara Jehi, Kees Braun

**Affiliations:** ^1^ Epilepsy Center Cleveland Clinic Cleveland OH USA; ^2^ Dept. of Neurology & Neurosurgery University Medical Center Utrecht Utrecht University the Netherlands

**Keywords:** cognitive outcomes, epilepsy surgery, histopathology, prognosis, seizure outcomes

## Abstract

Multiple factors influence the outcomes of epilepsy surgery. Prognostic indicators varying from clinical characteristics, imaging findings, ictal, and interictal electrophysiological activity have been linked to surgical outcomes. In this review, we focus on the relatively under‐studied role of the underlying epilepsy histopathology in driving post‐surgical outcomes, specifically focusing on the broad categories of seizure outcomes and cognitive outcomes. For each of these two outcomes of interest, we answer two questions: 1)‐ does etiology matter? and 2)‐ how could it matter? The goal is to review the existing literature on the relationship between etiology and surgical outcomes to provide the best possible judgment as to whether a causal relationship exists between histopathology and the ultimate surgical outcome as an initial step. Then, we delve into the possible mechanisms via which such relationships can be explained. We conclude with a call to action to the epilepsy surgery and histopathology research community to push the mechanistic understanding of the pathology‐outcome interaction and identify actionable knowledge and biomarkers that could inform patient care in a timely fashion.

## Abstract 2

In this review, we focus on the relatively under‐studied role of the underlying epilepsy histopathology in driving post‐surgical outcomes, specifically focusing on the broad categories of seizure outcomes and cognitive outcomes. For each of these two outcomes of interest, we answer two questions: (1) does etiology matter? and (2) how could it matter? We then delve into the mechanisms of these answers.

## 1 INTRODUCTION

Resective brain surgery is the most effective treatment for drug‐resistant focal epilepsy with seizure freedom rates ranging from 60% to 80% at one to two postoperative years, and 40% to 50% at 10 postoperative years (1). Additional benefits of surgery include longer life expectancy (2), lower risk of sudden death (3), better quality of life (4), improved mood (4), and recovered cognitive function/developmental trajectory (particularly in children) (5). Altogether, these outcomes are far superior to the alternative options of neuromodulation, ablation, or ongoing medical therapy. It is, therefore, critical to understand outcome determinants so we can better perfect our treatment.

Interest in studying surgical outcomes is in fact growing: A PubMed search on “epilepsy surgery outcomes” resulted in 6125 peer‐reviewed papers published from 2000 to 2020, with two‐third of these in the past decade. Dozens of surgical outcome predictors have been identified. All essentially converge on this central tenet: an epileptogenic zone, based on a structural lesion that is well‐visualized and restricted in its extent offers the best chance of post‐operative seizure freedom when completely resected. Research has, therefore, mainly focused on better localization of the epileptogenic lesion and zone, including finer imaging, sophisticated electrophysiology, and tools that combine both imaging and electrophysiology (such as magneto‐encephalography and EEG‐fMRI). In contrast, research of the underlying histopathological epilepsy substrate—the etiology/root cause which drives the visualization and extent of the epileptogenic zone—is relatively sparse.

In this review, we evaluate the data on the relationship between etiology and surgical outcomes, focusing on the two major outcome categories of seizure‐freedom, and cognitive/developmental outcomes, and highlight the possible mechanisms of those interactions.

## 2 SEIZURE OUTCOMES AND ETIOLOGY

### 2.1 Does etiology matter for seizure freedom?

Several studies have documented the correlation between the histopathological substrate and seizure‐freedom after epilepsy surgery. The most recent and largest scale such study included 9147 patients from 18 European countries, of whom seizure outcomes were available for 8191 (89·5%) participants at 2 years, and for 5577 (61·0%) at 5 years. The histopathological diagnoses of low‐grade epilepsy‐associated neuro‐epithelial tumor (LEAT), vascular malformation, and hippocampal sclerosis had the best seizure outcome at 2 postoperative years, with 77·5% of patients free from disabling seizures for LEAT, 74·0% for vascular malformation, and 71·5% for hippocampal sclerosis. The worst seizure outcomes at 2 years were seen for patients with focal cortical dysplasia type I or mild malformation of cortical development (50·0% free from disabling seizures), those with the malformation of cortical development‐other (52·3% free from disabling seizures), and for those with no histopathological lesion (53·5% free from disabling seizures) (6). These findings reproduce extensive prior literature consistently demonstrating the following:

1. *Any specific histopathological abnormality on resected presumably epileptic tissue is better than no histopathological lesion or nonspecific gliosis*. In a longitudinal study of 371 patients who had an anterior temporal lobectomy, 44% of cases who only had gliosis were seizure‐free 8 years after surgery, compared to 64% if a specific pathologic diagnosis was identified (7). Similarly, a systematic review and meta‐analysis of outcomes and their predictors after frontal lobe epilepsy surgery identified a lesional epilepsy origin as a significant predictor of long‐term seizure freedom (relative risk [RR] 1.67, 95% CI 1.36–28.6) (8). “Lesional epilepsy origin” in this context included histopathological findings of tumors, cortical dysplasia, or other focal lesions (52.2%–54.4% seizure‐free), as well as those with post‐traumatic epilepsy (60% seizure‐free), whereas patients with post‐infectious or other nonlesional/idiopathic epilepsies had less favorable outcomes (21.1%–32.7% seizure‐free) (8).
2. *Once a specific pathologic abnormality is identified though, and save for few exceptions, it is not entirely clear that the nature of the pathologic abnormality is critically relevant for seizure outcomes*. This is most obvious in the context of temporal lobe epilepsy (TLE) surgery. Let us first consider lesional TLE. The prospective multicenter Epilepsy Surgery study found in 2005 that 75% of patients with unilateral hippocampal sclerosis (HS) and a mesial temporal resection were seizure‐free, as opposed to 55% otherwise (including non‐lesional cases), suggesting that HS offers a favorable prognostic connotation (9). However, recent data suggest that such a favorable prognostic significance is actually conferred by *any* unilateral temporal MRI lesion, and not necessarily by HS, especially with concordant ictal and interictal EEG findings (1). In fact, while using the new international consensus classification for HS proposed by the International League against Epilepsy (10) demonstrated a differential influence of hippocampal subfields to memory formation (11), a credible parallel significance in a differential influence on seizure outcomes remains to be defined. Let us consider surgery in the context of a hippocampus that appears normal on brain MRI. A recent study publishing a nomogram and an online risk calculator for individualized prediction of seizure and naming outcome found clear additional value in including surgical histopathology in outcome calculation, but did not identify any incremental discriminatory value in classifying that histopathology beyond a distinction of “normal” to “abnormal” (12). In the context of frontal lobe surgery, seizure outcomes are similarly favorable with LEATs, Type II focal cortical dysplasia, and other lesions (8, 13). No correlation was found between focal cortical dysplasia (FCD) subtype as defined by the revised ILAE histopathology classification (14) and seizure outcome in some studies (15), while others suggested poorer outcomes with FCD IIIa (16), FCD Type I (17), and mMCD (18).

### 2.2 How could etiology matter for seizure outcome?

*Pathology drives imaging findings that guide and influence the success of epilepsy surgery*: Surgical histopathology routinely correlates with seizure outcomes on univariate analysis in outcome research studies, but rarely retains its significance after multivariate analysis, except when considered under the major classifications of “normal” versus “abnormal,” or when only a few con‐founders are considered, as was the case in the recent European Epilepsy Brain Bank study (19). This suggests that with our current understanding and classification of histopathology, this variable exerts its prognostic value through another more obvious patient characteristic: the most likely candidate is neuroimaging. The most favorable histopathological substrates (tumors, hippocampal sclerosis, type IIb cortical dysplasia) typically produce MRI visible lesions that are easy to delineate and resect, while the most challenging histopathological substrates (FCD Type I, and non‐specific gliosis) do not have an equally obvious imaging border, leading to more challenging definitions of the epileptogenic zone and thus poorer seizure outcomes.

*Histopathological abnormalities extend beyond the MRI‐visible lesion, further influencing the odds of postoperative seizure‐freedom*: The above attribution of all prognostic implications of pathology to its imaging signature would be an oversimplification—or at least an incomplete view—of the mechanisms by which histopathology influences seizure outcomes. One simply needs to consider situations where the epileptogenic zone (and by inference, the epileptic substrate) is either smaller or larger than the MRI‐visible lesion: the classical examples are multi‐lobar polymicrogyrias for the former where subtotal resections within the large malformation can lead to seizure freedom (20), and temporal cavernous malformations for the latter where the odds of postoperative seizure‐freedom are bolstered by including the hippocampus in addition to the cavernoma in the resection (21, 22). We would be remiss here not to mention the well‐documented observations of microscopic abnormal histopathology beyond the borders of the MRI‐visible lesion in the context of cortical dysplasia as another example of how a pathological substrate can create seizures without being visible on imaging (23). Altogether, these observations reinforce the idea that the spatial extent of the epileptogenic substrate (histopathology) is not always equivalent to the MRI‐visible lesion. On another less understood level, it is unclear whether different types of histopathological lesions in the same anatomical location can differentially modulate an epileptic network and lead to different extent of epileptogenicity beyond the lesional site, thus leading to different outcomes. This is a hypothetical consideration with no empiric support at this point.

*Molecular, genetically driven, biomarkers that may or may not be driven by histopathology are actually the outcome determinants*: The subgroup of patients with epilepsy whose seizures recur after an initial surgery, and subsequently undergo one or more re‐operations offer a unique window at understanding the role of pathology in driving seizure outcomes. The largest study of re‐operations evaluated 898 patients, including 110 who had reoperations of whom 92 had a total of two resective surgeries and 18 patients had three or more (24). Two years after the index (most recent) surgery, 69% of patients with no prior surgeries had an Engel score of I, as opposed to only 42% of those with one prior surgery, and 33% of those with two or more prior resections (*p* < 0.001). Among surgical outcome predictors, the number of prior epilepsy surgeries, female sex, lesional initial magnetic resonance imaging, no prior history of generalization, and pathology correlated with better seizure outcomes on univariate analysis. However, only sex (*p* = 0.011), history of generalization (*p* = 0.016), and number of prior surgeries (*p* = 0.002) remained statistically significant in the multivariate model. A detailed review of the 18 patients with three or more resective surgeries showed that the histopathological classification either remained the same across all resections or evolved from specific findings initially (HS or FCD) into non‐specific gliosis or “normal” classification with later resections (24). Ironically, the most refractory of patients—in the surgical sense—are those with no clear abnormalities on histopathology. This suggests a histopathology‐independent, potentially genetic mechanism, increasing the susceptibility to seizure‐recurrence in some individuals with epilepsy.

## 3 COGNITIVE OUTCOMES AND ETIOLOGY

### 3.1 Does etiology matter for cognitive outcome?

Most studies on cognitive outcome in adults who underwent epilepsy surgery address the risks and predictors of **cognitive decline**—in particular, memory after temporal lobe surgery (25, 26). In contrast, pediatric surgical series tend to focus primarily on developmental and **cognitive gains,** because surgery in children often aims to not only stop seizures but also improve development and restore cognitive progress (27, 28). In general, cognitive function and neurodevelopment of people with epilepsy are determined by the complex interplay between genetic, environmental, epilepsy‐, and treatment‐related variables (29). Many children with epilepsy, particularly those uncontrolled by antiseizure medication (ASM), have cognitive impairments and developmental delay. Disentangling the influence of contextual variables, genetic background, the epileptogenic pathology, the seizure disorder itself—with status epilepticus, epileptic encephalopathy, frequent seizures and interictal EEG discharges representing different degrees within a spectrum of severity—and the use of ASM, on eventual cognitive functioning of the child, is difficult, if not impossible (27). Epilepsy surgery stops the impact of seizures, epileptiform discharges, and ASM use, and may allow a catch‐up of the child's development, particularly so if there was a presurgical cognitive arrest or decline, in the context of epileptic encephalopathy (30).

The determinants of postoperative developmental outcome have been reviewed before (e.g: (30, 31, 32)). Although many factors have been reported to influence the cognitive outcome, results of associations were not unequivocal and multivariable analyses of sufficiently large cohorts are lacking (30). As an example; large hemispheric malformations of development often cause an early onset severe epileptic encephalopathy, that would not only lead to early surgery, but also to a relatively poor cognitive outcome. Univariate analysis of associations of outcome in a heterogenous cohort could then falsely suggest that a shorter epilepsy duration relates to poorer outcome, whereas the opposite is probably true for that particular subgroup of patients.

In this context, there is controversy regarding the importance of epileptogenic pathology for postoperative cognitive outcome. In adult studies, pathology was often not accounted for—or not identified—when assessing determinants of cognition, with only a few exceptions. For example, the absence of an MCD on preoperative MRI in frontal lobe surgery related to worsened performance IQ and visual delayed memory (33). In temporal lobe surgery, patients with dual pathology had a better cognitive outcome than those with hippocampal sclerosis alone (34), and the absence of a histopathological lesion correlated with more severe memory loss (35). Nevertheless, pathology did not contribute to the decline of naming or verbal memory in two recently developed prediction models of cognitive outcome following temporal lobe surgery (36, 37).

In children, only a few studies reported an **independent** influence of pathology. Postoperative change of developmental quotient (DQ) in a cohort of 115 children who underwent hemispherectomy revealed more postoperative DQ improvement in children with a hemispheric cortical dysplasia than in those with vascular lesions or other/miscellaneous pathologies. This difference remained significant after accounting for presurgical DQ. Eventual DQ, however, was not independently associated with pathology (38). Another single‐center study, including 100 children who underwent resective surgery and completed pre‐ and postoperative IQ assessment, found that pathology independently predicted outcome, with higher IQ in children with hippocampal sclerosis or tumors, compared to the malformations of cortical development. In this multivariable regression analysis, etiology was included next to age at onset, age at surgery, and extent of the epileptogenic focus (uni‐ vs. multilobar) (39). In one study, total IQ and language scores were reported to be lower after hemispherectomy in children with malformations of cortical development (MCD) than in vascular etiologies, but this was only tested using univariate analysis (40).

In the vast majority of studies, however, etiology was not identified as a predictor of cognitive outcome. Univariate analyses of children who underwent hemispherectomy (29), and of infants in the first 3 years of life who underwent hemispheric or resective surgery (41, 42), revealed no correlation between etiology and eventual cognitive outcome or change in IQ or DQ. Similarly; in a group of 42 children with FCD, subtypes were not related to eventual postoperative IQ (43). Multivariable analyses revealed that etiology was not independently related to postoperative IQ or DQ (38, 44, 45), to change in IQ/DQ (44, 45), or to postoperative verbal language outcome (46), after different types of epilepsy surgery.

Variables that did—independently and strongly—correlate with cognitive outcome were the duration of epilepsy (the shorter the better) (19, 38, 41, 43, 44, 47, 48, 49, 50), age at surgery (the older the better) (39), lesion extent (hemispheric and multilobar poorer than unilobar) (39, 41), post‐operative seizure control (see (30, 46)), contralateral MRI abnormalities in hemispherectomy (29, 46, 51), presence of epileptic encephalopathy (predicting more increase in IQ but more severe developmental delay) (42, 43), parental education (the higher the more increase in IQ) (45), and ASM withdrawal (correlating with higher eventual IQ and more gain in IQ) (45, 51, 52). Many of these factors also determined preoperative cognitive functioning, which is a known important and independent predictor of postoperative cognition (38, 41, 42, 44, 45, 53).

### 3.2 How could etiology matter for cognitive outcome?

From the studies reviewed above, it seems that epileptogenic pathology itself has little—if any—direct influence on cognitive outcome after pediatric epilepsy surgery. However, large individual participant data (IPD) meta‐analyses that include all of the above predictors would be required to appreciate its true independent predictive relevance. In patients with discrete epileptogenic lesions, located in identical brain areas, who undergo a complete resection after a similar epilepsy duration, and who reach both seizure‐ and drug‐freedom, the exact underlying pathology—for example, tumor versus cavernoma or type 2 FCD—may not be relevant for cognitive outcome. More important is probably the relation between the reversibility of functional disturbances outside the epileptogenic zone, and the cognitive functions that may have resided in the resected epileptogenic zone (25, 31). We know, from animal and human studies, that focal seizures can cause widespread changes in brain connectivity and networks, that may be irreversible and progressive with time and relate to cognitive functioning (for review, see (54)). Eventual outcome is determined by the structural integrity and the functional reserve of the “remaining brain” after surgery (25) and, thus, by the extent to which the brain has been exposed to seizures—that is, duration and severity of the active seizure disorder—and to drugs.

From this perspective, the underlying etiology can ***directly and independently*** relate to postoperative cognitive outcome only if it affects the brain outside the epileptogenic lesion that is resected. This can be the case in particular syndromes with multiple discrete lesions, such as tuberous sclerosis complex or multiple cavernomas. Similarly, seizure‐ and cognitive outcomes after hemispherectomy are poorer in patients with contralateral MRI abnormalities that are inherent to the epileptogenic etiology, such as bilateral perinatal vascular damage or developmental abnormalities that extend outside one hemisphere, for example, a reduced contralateral hemispheric volume in hemimegalencephaly. Finally, when resective epilepsy surgery is performed in the context of a monogenic disorder (55)—for example, FCD in DEPDC5 or other GATOR1‐related mutations, or hippocampal sclerosis as dual pathology in Dravet syndrome—cognitive outcome hypothetically not only depends on the epilepsy‐related variables, but also on the primary genetic defect.

***Indirectly*** , epileptogenic pathology may affect cognitive functioning if it is related to known other predictors of outcome. As an example, patients with mMCD or FCD type 1 may have a longer duration of epilepsy before surgery is performed, because their lesions are more often invisible on conventional MRI. Longer duration of their active epilepsy could lead to poorer outcome. In addition, patients with less discrete lesions carry a higher risk of incomplete resection and not reaching seizure‐freedom. This, and their ongoing ASM dependency, will affect cognitive outcome as well. Finally, some lesions in early life may predispose to an epileptic encephalopathy—for example, electrical status epilepticus in polymicrogyria—that, by itself, negatively influences the developmental outcome of the child.

## 4 CONCLUSION

At first sight, current literature suggests that pathology matters a lot in driving seizure outcomes and less in cognitive outcomes. The close correlation between pathology and other epilepsy‐related variables—for example, age, duration or severity of active epilepsy, MRI‐visibility—that largely contribute to surgical outcomes, complicates the interpretation of results of cohort studies that often included too little patients to appreciate independent predictive significance of pathology in multivariable analyses. The degree to which the brain is structurally and functionally affected outside the MRI‐visible and resected lesion may be an important determinant of both seizure and cognitive outcomes, much more so than the pathological diagnosis itself. It is a challenge to define the true epileptogenic substrate: that requires a resolution beyond our current histopathological classification. The prognostic value of pathology will remain in the academic and hypothetical frameworks until we identify molecular signatures that truly portend the equivalent of refractoriness to surgical therapy, or better still, markers that can offer direct actionable knowledge to drive treatment decisions at the point of care, as is done for example in the field of oncology.

## DATA AVAILABILITY STATEMENT

No data was used to generate this manuscript.

## References

[1] McGOvern R, Jehi L, Gnzalez‐Martinez JA, Bingaman W. Outcomes and complications of epilepsy surgery. In: Wyllie's treatment of epilepsy: principles and practice. 7th ed. Wolters Kluwer. Chapter 89. 2020:1012–26.

[2] Choi H, Sell RL, Lenert L, Muennig P, Goodman RR, Gilliam FG, et al. Epilepsy surgery for pharmacoresistant temporal lobe epilepsy: a decision analysis. JAMA. 2008;300(21):2497–505. 19050193 10.1001/jama.2008.771

[3] Sperling MR, Barshow S, Nei M, Asadi‐Pooya AA. A reappraisal of mortality after epilepsy surgery. Neurology. 2016;86(21):1938–44. 27164679 10.1212/WNL.0000000000002700

[4] Winslow J, Hu B, Tesar G, Jehi L. Longitudinal trajectory of quality of life and psychological outcomes following epilepsy surgery. Epilepsy Behav. 2020;111:107283. 10.1016/j.yebeh.2020.107283. 32759066 PMC8892470

[5] Kellermann TS, Wagner JL, Smith G, Karia S, Eskandari R. Surgical management of pediatric epilepsy: decision‐making and outcomes. Pediatr Neurol. 2016;64:21–31. 27568292 10.1016/j.pediatrneurol.2016.06.008

[6] Lamberink HJ, Otte WM, Blümcke I, Braun KPJ, Aichholzer M, Amorim I, et al. Seizure outcome and use of antiepileptic drugs after epilepsy surgery according to histopathological diagnosis: a retrospective multicentre cohort study. Lancet Neurol. 2020;19(9):748–57. 32822635 10.1016/S1474-4422(20)30220-9

[7] Jeha LE, Najm IM, Bingaman WE, Khandwala F, Widdess‐Walsh P, Morris HH, et al. Predictors of outcome after temporal lobectomy for the treatment of intractable epilepsy. Neurology. 2006;66:1938–40. 16801667 10.1212/01.wnl.0000219810.71010.9b

[8] Englot DJ, Wang DD, Rolston JD, Shih TT, Chang EF. Rates and predictors of long‐term seizure freedom after frontal lobe epilepsy surgery: a systematic review and meta‐analysis. J Neurosurg. 2012;116(5):1042–8. 22304450 10.3171/2012.1.JNS111620

[9] Spencer SS, Berg AT, Vickrey BG, Sperling MR, Bazil CW, Shinnar S, et al. Predicting long‐term seizure outcome after resective epilepsy surgery: the multicenter study. Neurology. 2005;65(6):912–8. 16186534 10.1212/01.wnl.0000176055.45774.71

[10] Blümcke I, Thom M, Aronica E, Armstrong DD, Bartolomei F, Bernasconi A, et al. International consensus classification of hippocampal sclerosis in temporal lobe epilepsy: a Task Force report from the ILAE Commission on Diagnostic Methods. Epilepsia. 2013;54(7):1315–29. 23692496 10.1111/epi.12220

[11] Coras R, Pauli E, Li J, Schwarz M, Rössler K, Buchfelder M, et al. Differential influence of hippocampal subfields to memory formation: insights from patients with temporal lobe epilepsy. Brain. 2014;137(Pt 7):1945–57. 24817139 10.1093/brain/awu100

[12] Morita‐Sherman M, Louis S, Vegh D, Busch RM, Ferguson L, Bingaman J, et al. Outcomes of resections that spare vs remove an MRI‐normal hippocampus. Epilepsia. 2020;61(11):2545–57. 33063852 10.1111/epi.16694 PMC7879196

[13] Simasathien T, Vadera S, Najm I, Gupta A, Bingaman W, Jehi L, et al. Improved outcomes with earlier surgery for intractable frontal lobe epilepsy. Ann Neurol. 2013;73(5):646–54. 23494550 10.1002/ana.23862

[14] Blümcke I, Thom M, Aronica E, Armstrong DD, Vinters HV, Palmini A, et al. The clinicopathologic spectrum of focal cortical dysplasias: a consensus classification proposed by an ad hoc Task Force of the ILAE Diagnostic Methods Commission. Epilepsia. 2011;52(1):158–74. 21219302 10.1111/j.1528-1167.2010.02777.x PMC3058866

[15] Mühlebner A, Gröppel G, Dressler A, Reiter‐Fink E, Kasprian G, Prayer D, et al. Epilepsy surgery in children and adolescents with malformations of cortical development–outcome and impact of the new ILAE classification on focal cortical dysplasia. Epilepsy Res. 2014;108(9):1652–61. 25219355 10.1016/j.eplepsyres.2014.08.012

[16] Johnson AM, Sugo E, Barreto D, Cunningham AM, Hiew C‐C, Lawson JA, et al. Clinicopathological associations in temporal lobe epilepsy patients utilising the current ILAE focal cortical dysplasia classification. Epilepsy Res. 2014;108(8):1345–51. 25048307 10.1016/j.eplepsyres.2014.06.013

[17] See S‐J, Jehi LE, Vadera S, Bulacio J, Najm I, Bingaman W, et al. Surgical outcomes in patients with extratemporal epilepsy and subtle or normal magnetic resonance imaging findings. Neurosurgery. 2013;73(1):68–76. 23615090 10.1227/01.neu.0000429839.76460.b7

[18] Veersema TJ, Swampillai B, Ferrier CH, Eijsden P, Gosselaar PH, van Rijen PC, et al. Long‐term seizure outcome after epilepsy surgery in patients with mild malformation of cortical development and focal cortical dysplasia. Epilepsia Open. 2019;4(1):170–5. 30868127 10.1002/epi4.12289 PMC6398095

[19] Ko A, Kim SH, Kim SH, Park EK, Shim K‐W, Kang H‐C, et al. Epilepsy surgery for children with low‐grade epilepsy‐associated tumors: factors associated with seizure recurrence and cognitive function. Pediatr Neurol. 2019;91:5–56. 10.1016/j.pediatrneurol.2018.10.008 30477743

[20] Maillard LG, Tassi L, Bartolomei F, Catenoix H, Dubeau F, Szurhaj W, et al. Stereoelectroencephalography and surgical outcome in polymicrogyria‐related epilepsy: a multicentric study. Ann Neurol. 2017;82(5):781–94. 29059488 10.1002/ana.25081

[21] Englot DJ, Han SJ, Lawton MT, Chang EF. Predictors of seizure freedom in the surgical treatment of supratentorial cavernous malformations. J Neurosurg. 2011;115(6):1169–74. 21819194 10.3171/2011.7.JNS11536

[22] Jehi LE, Palmini A, Aryal U, Coras R, Paglioli E. Cerebral cavernous malformations in the setting of focal epilepsies: pathological findings, clinical characteristics, and surgical treatment principles. Acta Neuropathol. 2014;128(1):55–65. 24831066 10.1007/s00401-014-1294-y

[23] Widdess‐Walsh P, Jeha L, Nair D, Kotagal P, Bingaman W, Najm I, et al. Subdural electrode analysis in focal cortical dysplasia: predictors of surgical outcome. Neurology. 2007;69(7):660–7. 17698787 10.1212/01.wnl.0000267427.91987.21

[24] Yardi R, Morita‐Sherman ME, Fitzgerald Z, Punia V, Bena J, Morrison S, et al. Long‐term outcomes of reoperations in epilepsy surgery. Epilepsia. 2020;61(3):465–78. 32108946 10.1111/epi.16452 PMC7224413

[25] Baxendale S. The cognitive costs, contraindications and complications of epilepsy surgery in adults. Curr Opin Neurol. 2020;33:207–12. 32073438 10.1097/WCO.0000000000000799

[26] Dulay M, Busch R. Prediction of neuropsychological outcome after resection of temporal and extratemporal seizure foci. Neurosurg Focus. 2012;32(3):E4. 10.3171/2012.1.FOCUS11340 22380858

[27] Braun KPJ. Influence of epilepsy surgery on developmental outcomes in children. Eur J Paediatr Neurol. 2020;24:40–2. 31917082 10.1016/j.ejpn.2019.12.014

[28] Ryvlin P, Cross H, Rheims S. Epilepsy surgery in children and adults. Lancet Neurol. 2014;13:1114–26. 25316018 10.1016/S1474-4422(14)70156-5

[29] Boshuisen K, van Schooneveld MMJ, Leijten FSS, de Kort GAP, van Rijen PC, Gosselaar PH, et al. Contralateral MRI abnormalities affect seizure and cognitive outcome after hemispherectomy. Neurology. 2010;75:1623–30. 21041785 10.1212/WNL.0b013e3181fb4400

[30] Van Schooneveld MM, Braun KP Cognitive outcome after epilepsy surgery in children. Brain Dev. 2013;35:721–9. 23434294 10.1016/j.braindev.2013.01.011

[31] Moosa AN, Wyllie E. Cognitive outcome after epilepsy surgery in children. Semin Pediatr Neurol. 2017;24:331–9. 29249513 10.1016/j.spen.2017.10.010

[32] Ramantani G, Reuner R. Cognitive development in pediatric epilepsy surgery. Neuropediatrics. 2018;49:93–102. 29207404 10.1055/s-0037-1609034

[33] Busch RM, Floden DP, Ferguson L, Mahmoud S, Mullane A, Jones S, et al. Neuropsychological outcome following frontal lobectomy for pharmacoresistant epilepsy in adults. Neurology. 2017;88:692–700. 28087827 10.1212/WNL.0000000000003611 PMC5317375

[34] Prayson BE, Prayson RA, Kubu CS, Bingaman W, Najm IM, Busch RM, et al. Effects of dual pathology on cognitive outcome following left anterior temporal lobectomy for treatment of epilepsy. Epilepsy Behav. 2013;28(3):426–31. 23886584 10.1016/j.yebeh.2013.05.040

[35] Helmstaedter C, Petzold I, Bien C. The cognitive consequence of resecting nonlesional tissues in epilepsy surgery—Results from MRI‐ and histopathology negative patients with temporal lobe epilepsy. Epilepsia. 2011;52(8):1402–8. 21740419 10.1111/j.1528-1167.2011.03157.x

[36] Busch RM, Hogue O, Kattan MW, Hamberger M, Drane DL, Hermann B, et al. Nomograms to predict naming decline after temporal lobe surgery in adults with epilepsy. Neurology 2018;91(23):e2144–e2152. 30404781 10.1212/WNL.0000000000006629 PMC6282231

[37] Ljunggren S, Andersson‐Roswall L, Imberg H, Samuelsson H, Malmgren K. Predicting verbal memory decline following temporal lobe resection for epilepsy. Acta Neurol Scand. 2019;140(5):312–9. 31273754 10.1111/ane.13146

[38] Jonas R, Nguyen S, Hu B, Asarnow RF, LoPresti C, Curtiss S, et al. Cerebral hemispherectomy: hospital course, seizure, developmental, language, and motor outcomes. Neurology. 2004;62:1712–21. 15159467 10.1212/01.wnl.0000127109.14569.c3

[39] Puka K, Rubinger L, Chan C, Smith ML, Widjaja E. Predictors of intellectual functioning after epilepsy surgery in childhood: the role of socioeconomic status. Epilepsy Behav. 2016;62:35–9. 27448241 10.1016/j.yebeh.2016.06.023

[40] Pulsifer MB, Brandt J, Salorio CF, Vining EPG, Carson BS, Freeman JM, et al. The cognitive outcome of hemispherectomy in 71 children. Epilepsia. 2004;45:243–54. 15009226 10.1111/j.0013-9580.2004.15303.x

[41] Kadish NE, Bast T, Reuner G, Wagner K, Mayer H, Schubert‐Bast S, et al. Epilepsy surgery in the first 3 years of life: predictors of seizure freedom and cognitive development. Neurosurgery. 2019;84:E368–E377. 30137548 10.1093/neuros/nyy376

[42] Loddenkemper T, Holland KD, Stanford LD, Kotagal P, Bingaman W, Wyllie E, et al. Developmental outcome after epilepsy surgery in infancy. Pediatrics. 2007;119:930–5. 17473093 10.1542/peds.2006-2530

[43] Veersema TJ, van Schooneveld MMJ, Ferrier CH, van Eijsden P, Gosselaar PH, van Rijen PC, et al. Cognitive functioning after epilepsy surgery in children with mild malformation of cortical development and focal cortical dysplasia. Epilepsy Behav. 2019;94:209–15. 30974349 10.1016/j.yebeh.2019.03.009

[44] D'Argenzio L, Colonnelli MC, Harrison S, Jacques TS, Harkness W, Vargha‐Khadem F, et al. Cognitive outcome after extratemporal epilepsy surgery in childhood. Epilepsia. 2011;52:1966–72. 22032791 10.1111/j.1528-1167.2011.03272.x

[45] Meekes J, van Schooneveld MMJ, Braams OB, Jennekens‐Schinkel A, van Rijen PC, Hendriks MPH, et al. Parental education predicts change in intelligence quotient after childhood epilepsy surgery. Epilepsia. 2015;56:599–607. 25705968 10.1111/epi.12938

[46] Moosa ANV, Jehi L, Marashly A, Cosmo G, Lachhwani D, Wyllie E, et al. Long‐term functional outcomes and their predictors after hemispherectomy in 115 children. Epilepsia. 2013;54:1771–9. 23980759 10.1111/epi.12342

[47] Basheer SN, Connolly MB, Lautzenhiser A, Sherman EMS, Hendson G, Steinbok P, et al. Hemispheric surgery in children with refractory epilepsy: seizure outcome, complications, and adaptive function. Epilepsia. 2007;48:133–40. 17241220 10.1111/j.1528-1167.2006.00909.x

[48] Delalande O, Bulteau C, Dellatolas G, Fohlen M, Jalin C, Buret V, et al. Vertical parasagittal hemispherectomy: surgical procedures and clinical long‐term outcomes in a population of 83 children. Neurosurgery. 2007;60[ONS Suppl 1]:ONS‐19–32. 10.1227/01.NEU.0000249246.48299.12 17297362

[49] Freitag H, Tuxhorn I. Cognitive function in preschool children after epilepsy surgery: rationale for early intervention. Epilepsia. 2005;46:561–7. 15816951 10.1111/j.0013-9580.2005.03504.x

[50] Thomas SG, Daniel RT, Chacko AG, Thomas M, Russell PSS. Cognitive changes following surgery in intractable hemispheric and sub‐hemispheric pediatric epilepsy. Childs Nerv Syst. 2010;26:1067–73. 20179944 10.1007/s00381-010-1102-5

[51] Boshuisen K, van Schooneveld MMJ, Uiterwaal CSPM, Cross JH, Harrison S, Polster T, et al. Intelligence quotient improves after antiepileptic drug withdrawal following pediatric epilepsy surgery. Ann Neurol. 2015;78:104–14. 25899932 10.1002/ana.24427

[52] Skirrow C, Cross JH, Cormack F, Harkness W, Vargha‐Khadem F, Baldeweg T, et al. Long‐term Intellectual outcome after temporal lobe surgery in childhood. Neurology. 2011;76:1330–7. 21482948 10.1212/WNL.0b013e31821527f0 PMC3090063

[53] Puka K, Tavares TP, Smith ML Development of intelligence 4 to 11 years after paediatric epilepsy surgery. J Neuropsychol. 2017;11:161–73. 26184054 10.1111/jnp.12081

[54] Braun KPJ. Preventing cognitive impairment in children with epilepsy. Curr Opin Neurol. 2017;30:140–7. 28141740 10.1097/WCO.0000000000000424

[55] Stevelink R, Sanders MWCB, Tuinman MP, Brilstra EH, Koeleman BPC, Jansen FE, et al. Epilepsy surgery for patients with genetic refractory epilepsy: a systematic review. Epileptic Disord. 2018;20:99–115. 29620010 10.1684/epd.2018.0959

